# From Radical Resection to Precision Surgery: Integrating Diagnostic Biomarkers, Radiomics-Based Predictive Models, and Perioperative Systemic Therapy in Head and Neck Oncology †

**DOI:** 10.3390/diagnostics16010049

**Published:** 2025-12-23

**Authors:** Luiz P. Kowalski, Carol R. Bradford, Jonathan J. Beitler, Juan Pablo Rodrigo, Orlando Guntinas-Lichius, Petra Ambrosch, Arlene A. Forastiere, Karthik N. Rao, Marc Hamoir, Nabil F. Saba, Alvaro Sanabria, Primoz Strojan, Kevin Thomas Robbins, Alfio Ferlito

**Affiliations:** 1 Head and Neck Surgery Department and LIM 28, University of São Paulo Medical School and A C Camargo Cancer Center, São Paulo 05403-000, Brazil; 2 Department of Otolaryngology-Head and Neck Surgery, College of Medicine, The Ohio State University, Columbus, OH 43210, USA; carol.bradford@osumc.edu; 3 Department of Radiation Oncology, Emory University School of Medicine and Atlanta Veterans Affairs Medical Center, Atlanta, GA 30322, USA; jonathan.beitler@va.gov; 4 Department of Otolaryngology-Head and Neck Surgery, Hospital Universitario Central de Asturias, University of Oviedo, ISPA, IUOPA, CIBERONC, 33011 Oviedo, Spain; jprodrigo@uniovi.es; 5 Department of Otorhinolaryngology, Head and Neck Surgery, Jena University Hospital, 07747 Jena, Germany; orlando.guntinas@med.uni-jena.de; 6 Department of Otorhinolaryngology, Head and Neck Surgery, University Hospital Schleswig-Holstein (UKSH), University of Kiel, 24105 Kiel, Germany; ambrosch@hno.uni-kiel.de; 7 Department of Oncology, School of Medicine, Johns Hopkins University, Baltimore, MD 21231, USA; af@jhmi.edu; 8 Department of Head and Neck Oncology, Sri Shankara Cancer Hospital and Research Center, Bangalore 560004, India; karthik.nag.rao@gmail.com; 9 Department of Otorhinolaryngology, Head and Neck Surgery, King Albert II Cancer Institute, Cliniques Universitaires Saint-Luc, Institut de Recherche Expérimentale, UC Louvain Medical School, 1200 Brussels, Belgium; marc.hamoir@saintluc.uclouvain.be; 10 Department of Hematology and Medical Oncology, Winship Cancer Institute, Emory University, Atlanta, GA 30322, USA; nfsaba@emory.edu; 11 Department of Surgery, Universidad de Antioquia, Medellin 050010, Colombia; alvarosanabria@gmail.com; 12 Department of Radiation Oncology, Institute of Oncology Ljubljana, Faculty of Medicine, University of Ljubljana, 1000 Ljubljana, Slovenia; pstrojan@onko-i.si; 13 Department of Otolaryngology Head and Neck Surgery, School of Medicine, Southern Illinois University, Springfield, IL 62794-9620, USA; kthomasrobbins@gmail.com; 14 International Head and Neck Scientific Group, 33100 Padua, Italy; profalfioferlito@gmail.com

**Keywords:** head and neck cancer, surgery, diagnostic biomarkers, predictive models, radiomics, comprehensive genomic profiling, tumor mutational burden, neoadjuvant immunotherapy, multidisciplinary care, precision medicine

## Abstract

Head and neck cancer surgery has evolved from radical organ-sacrificing procedures to function-preserving approaches integrated within multidisciplinary frameworks. This comprehensive literature review, concentrating on studies from the past five years while incorporating relevant publications from the last three decades and landmark historical papers, examines the evolving role of surgery emphasizing diagnostic methodologies including comprehensive genomic profiling, validated imaging biomarkers, and their clinical integration for treatment selection and response prediction. Modern surgical practice demonstrates a paradigm shift toward precision medicine through validated diagnostic technologies. Comprehensive genomic profiling identifies clinically actionable alterations in over 90% of head and neck squamous cell carcinomas, with tumor mutational burden serving as a validated predictive biomarker for immunotherapy response. Programmed death-ligand 1 (PD-L1) combined positive score functions as a validated diagnostic biomarker for immunotherapy efficacy, demonstrating significant clinical benefit in biomarker-selected populations. Radiomics-based predictive models utilizing machine learning algorithms achieve diagnostic accuracies exceeding 85% for treatment response prediction when validated across independent cohorts. Quantitative ultrasound spectroscopy combined with magnetic resonance imaging radiomics demonstrates high sensitivity and specificity for radiation response prediction. Habitat imaging techniques characterizing tumor microenvironmental heterogeneity predict pathologic complete response to neoadjuvant chemoimmunotherapy with area under the curve values approaching 0.90 in validation studies. Integration of these diagnostic methodologies enables response-adaptive treatment strategies, with neoadjuvant chemotherapy facilitating mandibular preservation and adjuvant therapy omission in over half of human papillomavirus (HPV)-associated cases following surgical downstaging. Clinical validation of these diagnostic platforms enables accurate treatment response prediction and informed surgical decision-making, though standardization across institutions and demonstration of survival benefits through prospective trials remain essential for broader implementation.

## 1. Introduction

Head and neck cancers represent a significant global health burden, with over 900,000 new cases and approximately 460,000 deaths annually worldwide, ranking among the most common malignancies globally. Head and neck malignancies encompass a heterogeneous group of cancers affecting anatomical regions critical for speech, swallowing, respiration, and facial aesthetics. Contemporary diagnostic evaluation relies on advanced imaging modalities with established high diagnostic accuracy, including magnetic resonance imaging (MRI) for superior soft tissue characterization, positron emission tomography/computed tomography (PET/CT) for metabolic assessment and nodal staging, and integrated PET/MRI combining anatomical detail with functional information, as validated through recent high-quality meta-analyses. The surgical management of these tumors has undergone fundamental transformation since the early 20th century, when radical en bloc resections represented the primary therapeutic approach [1]. This evolution reflects advances in surgical technique, improved understanding of tumor biology through molecular diagnostics, successful integration of perioperative chemotherapy and immunotherapy, development of validated imaging biomarkers, and growing recognition of the importance of functional preservation in cancer treatment [2,3,4,5,6,7,8,9,10,11].

Contemporary management exemplifies the integration of multiple disciplines working toward unified therapeutic goals within frameworks that accommodate diverse global healthcare environments [2,3]. Surgery, once performed in relative isolation with emphasis solely on oncologic control, now represents one component of carefully coordinated multidisciplinary treatment paradigms that prioritize both cancer cure and functional preservation according to patient preferences. This transformation evolved from landmark clinical research demonstrating that organ preservation strategies achieve equivalent survival outcomes while maintaining physiologic function in appropriately selected patients [4,5].

The modern surgeon navigates an increasingly complex therapeutic landscape driven by advances in molecular genetics, immunotherapeutics, and precision diagnostics. Two transformative developments in the past two decades include the identification of HPV as a causative factor for oropharyngeal cancer—a biologically distinct disease with superior prognosis compared to HPV-negative tumors [12]—and the development of immunotherapy with PD-1/PD-L1 expression serving as validated predictive biomarkers [13]. The integration of comprehensive genomic profiling platforms and quantitative imaging analytics has transformed surgical planning, enabling precision patient selection through tumor mutational burden assessment, immune microenvironment characterization, and radiomics-based response prediction. Recent clinical trials demonstrate the potential for diagnostic biomarker-driven treatment selection and neoadjuvant immunotherapy to fundamentally alter surgical approaches, necessitating critical evaluation of established practices and adaptive integration of emerging evidence [3,6,11].

## 2. Historical Evolution and Paradigm Shifts

### 2.1. The Era of Radical Surgery (19th Century–1970s)

The foundational era was characterized by radical, open resections with wide margins and acceptance of significant functional loss. Theodore Billroth pioneered this approach, performing the first laryngectomy in Vienna in 1873. On January 17, 1888, Polish surgeon Franciszek Jawdyński (1851–1896) performed a successful radical en bloc neck dissection, subsequently publishing a detailed description in the Polish journal *Gazeta Lekarska*. The procedure incorporated all elements of modern neck dissection, removing lymphoareolar contents from mandible to clavicle, including the sternocleidomastoid muscle. The internal jugular vein was ligated both inferiorly and superiorly and included in the specimen. The patient tolerated carotid ligation and survived seven years, apparently disease-free [14].

In 1905, George Washington Crile (1864–1943) of Cleveland, Ohio, presented “On the Surgical Treatment of Cancer of the Head and Neck, With a Summary of One Hundred and Twenty-One Operations Performed Upon One Hundred and Five Patients” at the Southern Surgical and Gynecologic Association meeting, published in the society’s *Transactions*. In June 1906, he presented similar material, “Excision of Cancer of the Head and Neck, With Special Reference to the Plan of Dissection Based on One Hundred and Thirty-Two Operations,” at the 57th annual American Medical Association meeting. This paper, published in the *Journal of the American Medical Association*, had widespread impact and revolutionized head and neck cancer treatment [3,6,7].

Subsequently, the Memorial Hospital approach under Hayes Martin became the international standard, with his “Commando operation” for advanced oral cancers representing the epitome of radical surgical philosophy [6]. However, the significant morbidity and functional deficits associated with these approaches motivated the search for alternative strategies.

### 2.2. The Organ Preservation Movement (1980s–2000s)

Functional and cosmetic morbidity from radical surgery, combined with the recognized effectiveness of cisplatin-based chemotherapy, catalyzed the organ preservation movement, which fundamentally altered treatment paradigms [2,3]. The landmark Veterans Affairs laryngeal preservation trial represented a pivotal moment, demonstrating that organ preservation could be achieved without compromising survival in carefully selected patients treated by experienced multidisciplinary teams providing balanced case assessment, therapeutic expertise, follow-up, and timely surgical salvage [2,3,4,8]. Building upon this foundation, the RTOG 91-11 trial subsequently demonstrated superior laryngeal preservation rates with concurrent cisplatin and radiation compared to induction chemotherapy. Both sequential and concurrent approaches resulted in longer laryngectomy-free survival, with the highest organ preservation rates in the concurrent arm [2,5]. Integration of medical oncology into head and neck cancer care during this era established the foundation for modern multidisciplinary management.

### 2.3. The Era of Minimally Invasive Precision Surgery and Diagnostic Integration (2000s–Present)

**Figure 1:**
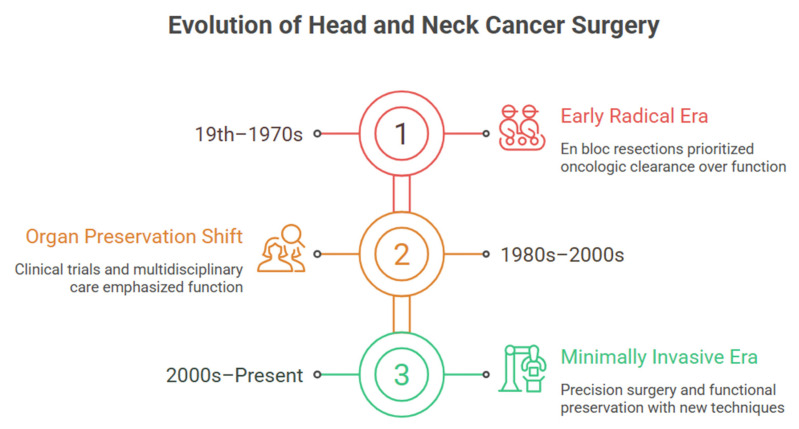
Evolution of head and neck cancer surgery integrating diagnostic biomarkers and precision medicine approaches.

Advances in CO_2_ laser technology, endoscopic optics, robotic manipulation, systemic therapy, and radiotherapy have enabled transoral resections without loss of locoregional control for selected upper aerodigestive tumors [3,6,9]. Concurrently, standardization of multidisciplinary tumor boards and perioperative collaboration became routine as treatment options expanded. Most significantly, integration of validated diagnostic biomarkers with neoadjuvant immunotherapy and chemotherapy to enable response-adaptive therapy is under intense investigation. Studies of perioperative immunotherapy in curative settings hold considerable promise. These strategies will fundamentally alter the standard of care, requiring sophisticated coordination between surgical, medical, and radiation oncology teams to optimize treatment sequencing and biomarker-driven patient selection (Figure 1) [10,11].

## 3. Multidisciplinary Care as the Foundation of Contemporary Practice

Although the head and neck region harbors diverse neoplastic entities including orbital tumors, sinonasal malignancies, salivary gland carcinomas, thyroid and parathyroid tumors, as well as bone, soft tissue, and cutaneous malignancies, this review focuses specifically on cancers arising in the oral cavity, oropharynx, and larynx, where squamous cell carcinoma represents the predominant histopathological variant, accounting for over 90% of cases. Histopathological confirmation through biopsy remains mandatory before therapeutic planning can proceed. Comprehensive clinical evaluation combined with advanced imaging modalities enables accurate disease extent assessment and precise oncological staging according to the Tumor-Node-Metastasis (TNM) classification system established by the Union for International Cancer Control (UICC) and the American Joint Committee on Cancer (AJCC), currently in its eighth edition. This standardized staging framework provides essential prognostic information and guides multidisciplinary treatment decision-making across diverse clinical scenarios.

Comprehensive preoperative evaluation integrates clinical, biochemical, and imaging assessments to determine surgical candidacy. Clinical evaluation encompasses tumor examination, cervical lymph node assessment, cranial nerve function, and airway patency. Biochemical workup includes complete blood count, renal and hepatic function, coagulation profile, and nutritional markers. Cross-sectional imaging with CT or MRI defines tumor extent and resectability, while PET/CT detects distant metastases and characterizes nodal disease. Cardiopulmonary assessment ensures adequate physiologic reserve for major surgery. This systematic framework enables patient selection, surgical planning, and multidisciplinary treatment sequencing.

Multidisciplinary care refers to coordinated team-based management wherein specialists collaboratively evaluate patients, develop integrated treatment plans, and provide synchronized care. The multidisciplinary tumor board serves as the operational framework where diagnostic findings and treatment options are discussed comprehensively, ensuring therapeutic recommendations reflect consensus while incorporating patient preferences and functional goals.

### 3.1. Evidence-Based Benefits of Integrated Care Models

Implementation of multidisciplinary tumor boards (MTBs) has emerged as a cornerstone of modern head and neck cancer management, with accumulating evidence supporting their clinical impact. Liu et al. demonstrated survival benefits following MTB implementation, with five-year disease-specific survival improving from 52% to 75% (*p* = 0.003) and overall survival increasing from 40% to 61% (*p* = 0.008). The study revealed that structured multidisciplinary evaluation facilitated coordination between specialties, resulting in improved outcomes even when controlled for stage and confounding variables [15].

Burkhardt et al. [16] demonstrated in a retrospective study that implementation of pretherapeutic MTB evaluation significantly enhanced adherence to evidence-based clinical guidelines, with compliance rates reaching 79.6% compared to 68% in non-MTB cohorts (*p* < 0.01). This improvement manifested particularly in appropriate utilization of neck dissection procedures, where MTB-reviewed cases showed substantially higher rates of comprehensive surgical intervention (90.5% versus 78.9%, *p* < 0.001). The multidisciplinary framework facilitates standardized treatment protocols while serving valuable educational and quality assurance functions within cancer centers.

However, these benefits must be weighed against notable limitations. MTB presentation introduces statistically significant delays in treatment initiation, with a mean difference of approximately 13 days between MTB and non-MTB groups (33.5 versus 20.1 days, *p* < 0.001). This temporal prolongation, observed consistently across all UICC stages, raises concerns given established evidence linking treatment delays to tumor progression and clinical upstaging. Perhaps most striking is the absence of measurable survival benefit in this particular study, which demonstrated no significant differences in three-year survival rates, disease-free survival, or five-year survival metrics between MTB and non-MTB cohorts, suggesting that enhanced guideline adherence does not necessarily translate into improved oncologic outcomes in oral cavity cancer patients [16].

The relationship between certification standards requiring mandatory MTBs and patient outcomes has been comprehensively evaluated in the large-scale WiZen (Effectiveness of Care in Certified Cancer Centers) comparative cohort study conducted in Germany. Schmitt et al. analyzed nationwide data from approximately 22 million adult statutory health insurance beneficiaries between 2009 and 2017, encompassing 11 cancer entities including head and neck cancer. For head and neck malignancies, 8173 patients received initial treatment in German Cancer Society (DKG)-certified centers compared to 44,576 in non-certified institutions. The study demonstrated that initial cancer treatment in certified centers—where both preoperative and postoperative MTB evaluation is mandated—was associated with improved overall survival across all examined cancer entities. For head and neck cancer, the adjusted hazard ratio was 0.94 (95% CI: 0.89–1.00, *p* = 0.038), corresponding to an absolute risk reduction of 1.79 months. The survival advantage remained consistent even after extensive adjustment for patient-level confounders (age, sex, distant metastases, comorbidities) and hospital-level variables (bed capacity, university status, ownership). Notably, sensitivity analyses revealed that this survival benefit tended to strengthen with increasing duration of certification, suggesting cumulative effects of sustained implementation of evidence-based multidisciplinary care protocols. These findings provide population-level evidence supporting integration of mandatory MTB evaluation within certified cancer center frameworks, despite procedural delays inherent to multidisciplinary review processes [17].

Mechanisms underlying MTB benefits extend beyond treatment selection to encompass comprehensive care coordination. Incorporation of speech-language pathology, nutrition, palliative care, and social work services within the MTB framework demonstrated tangible functional improvements, including reduced pretreatment gastrostomy rates (39% versus 26%, *p* = 0.03) in advanced disease patients and improved quality of life with dignified end-of-life care in critically ill patients [15]. These findings underscore the importance of integrated care models that address both oncologic and functional outcomes through systematic coordination of specialized expertise.

### 3.2. Contemporary Treatment Team Structure

Modern treatment teams typically include surgical oncologists, radiation oncologists, medical oncologists, pathologists, radiologists, speech-language pathologists, nutritionists, palliative care specialists, and psychosocial support specialists, each contributing specialized expertise to treatment planning and execution while working toward unified patient-centered goals. Within this collaborative framework, the role of the surgeon has evolved significantly beyond ablative procedures, now encompassing initial diagnosis and staging, treatment selection, pretreatment support, assessment during chemoradiation therapy, post-treatment evaluation, salvage surgery, and management of late effects [18,19]. However, institutions with well-established multidisciplinary teams remain limited; therefore, lack of this organizational structure for most patients seeking therapy represents a major obstacle in terms of access to adequate care (Table 1).

## 4. Contemporary Surgical Techniques and Evidence-Based Implementation

### 4.1. Transoral Laser Microsurgery: Precision Through Technology

Transoral laser microsurgery (TLM) has been a standard technique over the past three decades. It employs precise CO_2_ laser technology combined with operative microscopy or exoscopy to achieve accurate tumor resection with excellent hemostatic control [9,20]. Technological advances include development of micromanipulators allowing precise laser beam focusing, pulsed laser systems resulting in reduced thermal tissue damage, and specialized surgical laryngoscopes and microinstruments. Oncological precision was further improved through development of standardized methods for pathohistological processing of resection specimens. Perioperative morbidity of TLM is low, as tracheostomy can be avoided in most cases. Functional outcomes are significantly superior to open surgery, due to avoidance of transcervical approaches and superior preservation of tumor-free surrounding structures. A prospective multicenter trial on TLM for supraglottic carcinoma resection reported aspiration-free swallowing rates of 95% at 12 months and no long-lasting impact on voice- and swallowing-related quality of life [21].

Currently, TLM is widely accepted for early laryngeal and selected oropharyngeal carcinomas. With adequate surgical experience, TLM can replace open partial laryngectomy and, in selected cases with tumors accessible endoscopically and resectable with function preservation, carcinomas of the oral cavity, oropharynx, and hypopharynx can be resected. The combination of TLM with selective neck dissection and adjuvant radiation represents a treatment concept that achieves favorable oncological and functional results [22].

### 4.2. Transoral Robotic Surgery: Evidence-Based Guidelines and Expanding Applications

Transoral robotic surgery (TORS) has revolutionized surgical access to the oropharynx and supraglottic larynx through technological innovation [9,18]. A multi-institutional randomized clinical trial demonstrated that primary transoral surgery with reduced postoperative radiation therapy results in improved oncologic and functional outcomes in intermediate-risk HPV-positive oropharyngeal cancer [23]. Recent comprehensive guidelines from the American Society of Clinical Oncology have established evidence-based recommendations for TORS implementation in multidisciplinary care of patients with oropharyngeal cancer [18]. The robotic platform provides surgeons with three-dimensional endoscopic visualization, wristed instrumentation offering seven degrees of freedom, tremor filtration, and motion scaling capabilities that significantly enhance surgical precision.

Recent advances in robotic surgery technology have introduced single-port systems that promise to further reduce surgical invasiveness while maintaining precision [24]. A prospective multicenter phase 2 clinical trial evaluated a next-generation single-port robotic surgical system for TORS in 47 patients, demonstrating promising outcomes: all patients safely underwent transoral resection without conversion to open surgery, with no intraoperative complications and mean estimated blood loss of only 15.4 mL [25]. The single-port architecture offers three fully articulating 6-mm instruments and a flexible stereoendoscopic camera deployed through a compact 25-mm port.

### 4.3. Radical Surgical Approaches: Contemporary Indications

Radical surgical procedures remain essential for advanced malignancies where tumor extent precludes function-preserving approaches. Total laryngectomy addresses advanced laryngeal cancers with extensive cartilage invasion, creating permanent tracheostoma while enabling tracheoesophageal voice rehabilitation. Composite resections combine mandibulectomy with soft tissue ablation for bone-invasive tumors, requiring fibular free flap reconstruction to restore facial contour and masticatory function. Maxillectomy procedures address sinonasal malignancies, with prosthodontic rehabilitation through obturator placement enabling functional restoration. Radical neck dissection, removing all lymphatic levels with sacrifice of sternocleidomastoid muscle, internal jugular vein, and spinal accessory nerve, remains indicated for extensive nodal disease with extranodal extension. These procedures require extensive preoperative counseling regarding functional losses and comprehensive rehabilitation planning within multidisciplinary frameworks [26,27].

## 5. Evidence-Based Patient Selection Criteria

Contemporary guidelines establish comprehensive criteria for optimal patient selection. Patients with lateralized oropharyngeal cancer represent optimal TORS candidates, while adequate transoral exposure must be confirmed through careful assessment of mandibular arch width, interincisor opening, and absence of trismus. Contraindications include tumors requiring significant soft palate resection, involvement of adjacent parapharyngeal fat, and tumors that abut the hyoid bone or extend into extrinsic tongue musculature [18].

Recent advances incorporate free flap reconstruction techniques into transoral robotic surgical protocols, demonstrating significant potential for functional preservation in patients undergoing oropharyngeal tumor resection. Contemporary evidence suggests that combining minimally invasive robotic approaches with microsurgical reconstruction enables progressive restoration of oral intake capacity, particularly in patients who do not require adjuvant radiotherapy. Multidisciplinary treatment strategies must consider critical anatomical factors, as hypoglossal nerve involvement and postoperative radiation therapy have been identified as independent predictors of compromised long-term swallowing function. These findings underscore the importance of selective case selection and individualized rehabilitation protocols to optimize patient-reported functional outcomes following complex head and neck oncologic procedures [28].

It is widely accepted that the optimal indication for TLM is early laryngeal cancer, and that treatment of advanced carcinomas and carcinomas in other locations requires special expertise. Similarly to TORS, adequate exposure is a prerequisite for TLM. Contraindications include poor anatomical access (trismus, macroglossia, limited neck extension, or mandibular abnormalities preventing adequate laryngeal visualization), advanced-stage tumors (especially with cartilage invasion or extralaryngeal spread), and severe dysphagia or aspiration risk [29].

## 6. Diagnostic Biomarker Integration and Perioperative Systemic Therapy

### Validated Predictive Biomarkers for Immunotherapy Selection

The recent integration of immunotherapy into head and neck cancer treatment represents one of the most significant advances in oncology, with profound implications for surgical planning and multidisciplinary care coordination. The KEYNOTE-689 phase 3 trial represents a breakthrough in locally advanced head and neck squamous cell carcinoma management, introducing immunotherapy as first-line treatment and establishing new standards for diagnostic biomarker-guided therapy. This randomized study enrolled 714 surgical candidates, comparing the addition of neoadjuvant and adjuvant pembrolizumab to standard care versus standard care alone [11].

Clinical results were compelling across all analyzed populations according to programmed death ligand 1 (PD-L1) combined positive score (CPS) status: tumors expressing PD-L1 with CPS of 10 or more (CPS-10 population), participants whose tumors expressed PD-L1 with CPS of 1 or more (CPS-1 population), and all participants irrespective of CPS (total population). In patients with PD-L1 CPS ≥ 10, event-free survival at 36 months was 59.8% versus 45.9% (hazard ratio 0.66; *p* = 0.004). Most importantly for surgical planning, neoadjuvant pembrolizumab did not compromise surgical feasibility, with approximately 88% of participants completing surgery in both groups. Notably, less than 22% of female patients were included in each arm, and the majority of patients (>60%) had oral cavity as the primary tumor site. Event-free survival was not significantly different in the female subgroup (25/77 in pembrolizumab arm versus 23/74 in standard of care arm), suggesting possible gender differences in response. However, given limited female patient enrollment, no definitive conclusions can be drawn [11].

The validation of PD-L1 CPS as a predictive biomarker represents a significant advancement in precision oncology. This diagnostic methodology involves immunohistochemical assessment using the 22C3 antibody clone on formalin-fixed paraffin-embedded tumor specimens. The CPS is calculated as the number of PD-L1-positive cells (tumor cells, lymphocytes, macrophages) divided by total viable tumor cells, multiplied by 100. Clinical validation across multiple trials has established CPS thresholds with distinct predictive value, enabling rational patient selection for immunotherapy interventions [11,30].

No data from KEYNOTE-689 [11] regarding improvement in organ preservation have been reported, but the trial represents an encouraging first step. The relatively low proportion of major pathological responses in the pembrolizumab arm and lack of difference in locoregional event rates between groups indicate limited local effectiveness. Additionally, further refinement of treatment combinations is needed, as not all patients likely require 17 pembrolizumab cycles (nine months versus three months of therapy in the standard group), and the effectiveness of immunotherapy in recurrent disease, if used in primary treatment, could be compromised. To enhance organ preservation, additional trials with combination therapies including both immunotherapy and chemotherapy will likely be needed. It is important to await more mature data regarding pathologic responses to pembrolizumab. Another unresolved question concerns surgical technique—whether to base margins on initial tumor extent before neoadjuvant therapy or to determine margins considering residual disease extension.

Imamura et al. [31] presented a narrative review comparing two landmark phase III trials—KEYNOTE-689 [11] and NIVOPOSTOP [32]—evaluating perioperative immune checkpoint inhibition in resectable, locally advanced HNSCC. KEYNOTE-689, using perioperative pembrolizumab, demonstrated improved event-free survival (HR 0.73; 95% CI 0.58–0.92) with notable reductions in distant recurrence. In contrast, NIVOPOSTOP, which tested adjuvant nivolumab with postoperative chemoradiotherapy in high-risk patients, achieved improved disease-free survival (HR 0.76; 95% CI 0.60–0.98) and superior locoregional control. Treatment-related adverse events and adherence were more favorable in KEYNOTE-689, supporting feasibility of a broad perioperative strategy [31].

The trials highlight different approaches to timing—KEYNOTE-689 priming immunity before surgery and consolidating after, while NIVOPOSTOP countered the immunosuppressive impact of adjuvant chemoradiotherapy. Each approach has distinct advantages, and neither is universally superior; treatment selection should be individualized based on recurrence risk, PD-L1 expression, and clinical feasibility. Both studies show encouraging trends toward overall survival benefit, although data remain immature. Collectively, they represent major advances in immunotherapy within curative definitive treatment contexts. Future priorities include refining biomarker-driven patient selection, optimizing neoadjuvant regimens, developing response-adapted de-escalation strategies, and ensuring cost-effectiveness in real-world practice. Mature overall survival data from these trials are awaited, but it appears that the combination of adjuvant nivolumab with postoperative chemoradiotherapy provides a more streamlined and resource-efficient strategy, especially for high-risk patients [31].

## 7. Comprehensive Neoadjuvant Strategies: Chemotherapy, Chemoradiation, and Targeted Approaches

### 7.1. Neoadjuvant Chemotherapy Plus Surgery

Neoadjuvant chemotherapy followed by surgery represents a promising paradigm shift from traditional approaches, emphasizing systemic escalation coupled with locoregional de-escalation [10,33,34]. A comparative study of 55 patients treated with neoadjuvant chemotherapy plus surgery versus 142 patients receiving concurrent chemoradiation revealed superior five-year disease-free survival in the surgical approach (96.1% versus 67.6%, *p* = 0.01) while eliminating long-term feeding tube dependency [10]. However, this study has several limitations, including temporal bias with older methods used to treat chemoradiation patients. Additionally, groups were unbalanced, with surgical patients having less N2c nodal disease, more non-smokers (80% versus 27%), and more T4 disease in the non-surgical arm. Furthermore, regarding alcohol consumption, 93% of surgical patients were never or light alcohol users. There were also remarkable differences in comorbidity index.

In a phase II trial by Chaukar et al. [35], mandibular preservation was achieved in 16 of 34 patients (47%) who received neoadjuvant chemotherapy, compared with none in the upfront surgery arm. Disease-free survival and overall survival were similar between arms. Chemotherapy-related grade III and IV toxicities occurred in 41% and 32% of patients, respectively, in the neoadjuvant and upfront surgery arms. These findings suggest that neoadjuvant chemotherapy can enable organ preservation without compromising survival, meriting further validation in phase III trials.

The biological rationale for neoadjuvant approaches is particularly compelling in HPV-positive disease with excellent locoregional control [33]. Neoadjuvant protocols with docetaxel and cisplatin-based regimens have demonstrated pathologic complete response rates exceeding 40% at both primary and nodal sites, enabling subsequent surgical de-escalation [10].

### 7.2. Neoadjuvant Chemoradiation

Neoadjuvant chemoradiation combines radiation therapy (45–50 Gy) with concurrent platinum-based chemotherapy for borderline resectable tumors requiring cytoreduction. This approach enables surgical downstaging and potentially less extensive resections while maintaining adequate margins. However, treatment-related fibrosis may complicate surgical dissection and reconstruction, and radiation-induced tissue changes can obscure pathological assessment. Patient selection must weigh downstaging benefits against morbidity risks and treatment delays. Prospective comparative data remain limited, necessitating individualized decision-making within multidisciplinary frameworks [36,37].

### 7.3. Neoadjuvant Targeted Therapies in Thyroid Cancer

Advanced differentiated thyroid cancers with actionable mutations increasingly benefit from neoadjuvant targeted therapy. BRAF-mutated tumors with locally advanced disease may achieve substantial regression with selective BRAF inhibitors, potentially converting unresectable disease to surgical candidacy. RET-altered thyroid cancers demonstrate responsiveness to selective RET inhibitors. Multikinase inhibitors including lenvatinib and sorafenib achieve tumor volume reduction in radioiodine-refractory disease, with 8–12 weeks treatment duration enabling maximal cytoreduction. Critical considerations include managing treatment-related toxicities affecting surgical candidacy and coordinating optimal timing between medical oncology and surgical oncology to capitalize on tumor response [38,39,40,41].

## 8. Perioperative Care Coordination

The complexity of integrating advanced surgical care with other therapies has highlighted the critical importance of standardized perioperative care protocols. Development of evidence-based Enhanced Recovery After Surgery (ERAS) protocols for head and neck cancer surgery with free flap reconstruction represents a significant advancement in standardizing perioperative care, identifying 17 key elements including preoperative carbohydrate treatment, pharmacologic thromboprophylaxis, perioperative antibiotics, goal-directed fluid management, and early mobilization [42].

Beyond these standardized protocols, understanding the full spectrum of perioperative risks becomes essential when counseling patients and planning multimodal treatment strategies. Cardiovascular complications represent particularly important considerations in this patient population. In a systematic review and meta-analysis of 53 studies, Asarkar et al. [43] reported a global pooled incidence of myocardial infarction after head and neck cancer treatment of 1.7% (*n* = 85,948 cases). Incidence was lowest with surgery alone (1.2%) and higher with multimodality regimens including adjuvant therapy (2.7%) or chemoradiotherapy (2.3%). These data underscore the importance of comprehensive perioperative risk assessment and the need for coordinated care protocols addressing both oncologic and medical comorbidities when integrating surgery with systemic therapies.

Systematic postoperative management requires multidisciplinary coordination addressing surgical complications and functional rehabilitation. Early care focuses on airway management with structured tracheostomy decannulation protocols, free flap monitoring during the critical 72-h period, and wound surveillance for infections or fistula formation. Nutritional support through enteral feeding with progressive swallowing rehabilitation guided by speech-language pathologists enables return to oral nutrition. Multimodal pain control strategies balance adequate analgesia with opioid dependency avoidance. Long-term surveillance follows evidence-based protocols with clinical examination and imaging every 3–4 months during the first two years, transitioning to less frequent intervals. Functional assessments including speech, swallowing, and quality of life measures guide ongoing rehabilitation. Management of late effects including lymphedema, fibrosis, trismus, and osteoradionecrosis requires ongoing multidisciplinary vigilance and physical therapy interventions [44,45,46,47,48].

## 9. Precision Medicine Integration: Molecular Profiling and Advanced Diagnostic Imaging

### 9.1. Molecular Pathology: From Single Biomarkers to Comprehensive Genomic Landscapes

Integration of next-generation sequencing technologies has expanded the diagnostic armamentarium beyond traditional histopathological assessment. Comprehensive genomic profiling (CGP) has emerged as a powerful diagnostic tool for therapeutic stratification in head and neck squamous cell carcinoma. The diagnostic and therapeutic utility of CGP extends beyond disease characterization to prognostic stratification for immunotherapy and identification of trial-eligible molecular subsets [49].

Analysis of over 1100 head and neck cancer cases from national and institutional CGP databases demonstrates that high tumor mutational burden (TMB ≥10 mutations/megabase) in squamous cell carcinoma correlates with prolonged survival following immune checkpoint inhibitor therapy, whereas CCND1 amplification associates with inferior response. The diagnostic methodology for TMB assessment involves whole-exome or targeted panel sequencing with bioinformatic algorithms to quantify somatic mutations per megabase of coding DNA examined. Clinical validation studies have established TMB as a predictive biomarker across multiple tumor types, with standardized cutoff values enabling consistent diagnostic interpretation [49].

Critically, this large-scale genomic analysis revealed that 23% of sequenced cases qualified for biomarker-matched clinical trials and 5% received genomically directed therapy, underscoring CGP’s role in expanding treatment access beyond conventional options [49]. In prospective clinical implementation, actionable somatic alterations are identified in over 90% of patients undergoing CGP, with a median of four targetable genomic events per case, thereby generating concrete therapeutic leads for clinical trial enrollment and off-label use of approved targeted agents [50].

The diagnostic workflow for CGP typically involves (a) procurement of adequate tumor tissue (minimum 20% tumor content); (b) DNA extraction and quality assessment; (c) library preparation and next-generation sequencing; (d) bioinformatic analysis including mutation calling, copy number variation detection, and structural variant identification; (e) clinical interpretation by molecular pathologists; (f) integration with clinical data for treatment recommendations [51,52,53,54,55,56,57,58,59,60,61,62,63,64,65,66].

By quantifying TMB, delineating copy-number amplifications, and detecting rare oncogenic fusions not accessible through routine histopathology, CGP transforms tumor characterization from purely morphological classification into molecularly annotated profiles that inform immunotherapy candidacy, targeted therapy selection, and clinical trial eligibility. This integration of quantitative genomic metrics with traditional histopathology and PD-L1 expression represents a paradigm shift in patient stratification, enabling multidisciplinary teams to distinguish patients most likely to benefit from immunotherapy from those requiring alternative precision approaches [49,50].

Despite considerable optimism regarding the potential of HPV circulating tumor DNA diagnostics to enable safer treatment de-intensification strategies in HPV-associated malignancies, a prospective investigation revealed significant limitations in clinical application. The study evaluated the utility of circulating tumor-derived viral DNA quantification as a decision-making tool for adjuvant radiotherapy omission in surgically treated HPV-positive oropharyngeal carcinomas, with patients demonstrating undetectable postoperative tumor-tissue-modified viral DNA scores being observed without immediate adjuvant treatment. However, the surveillance strategy yielded an unacceptably high gross recurrence rate of 25%, with radiographic disease progression occurring independently of antecedent biomarker detection in the majority of cases. These findings demonstrate that molecular residual disease assessment using current HPV circulating tumor DNA platforms lacks sufficient sensitivity to safely guide treatment de-escalation decisions in this patient population [53].

This negative result underscores the critical importance of rigorous prospective validation before implementing novel diagnostic biomarkers in clinical practice. The diagnostic performance characteristics—particularly sensitivity for minimal residual disease detection—must be thoroughly established across diverse patient populations before biomarker-guided treatment modifications can be safely implemented.

### 9.2. Immune Microenvironment Characterization as Predictive Biomarker

Beyond genomic alterations, characterization of the immune microenvironment has emerged as a predictive diagnostic tool for neoadjuvant response. Preliminary investigations demonstrate that intratumoral CD103^+^ CD8^+^ T cell infiltration correlates with pathological response to chemoimmunotherapy in head and neck cancer cohorts [54]. These immune biomarkers hold promise for preoperative identification of patients most likely to achieve significant tumor regression, potentially informing decisions regarding organ preservation versus definitive resection and guiding the extent of surgical margins in post-neoadjuvant settings.

The diagnostic methodology involves multiplex immunohistochemistry or immunofluorescence on formalin-fixed paraffin-embedded tumor specimens, with quantitative image analysis to determine spatial distribution and density of immune cell populations. Standardization of these assays across institutions remains a priority, with ongoing efforts to establish reproducible protocols and interpretive criteria [55,56].

### 9.3. Imaging Innovations: Radiomics-Based Predictive Models and Diagnostic Validation

Parallel to molecular advances, imaging technologies have evolved from purely anatomical staging modalities to platforms capable of quantitative tumor phenotyping and response prediction. Radiomics—the high-throughput extraction of quantitative features from medical images—combined with machine learning algorithms, has demonstrated capacity to predict therapeutic response and metastatic risk with clinically actionable accuracy. Multimodal feature integration represents a particularly promising frontier in diagnostic imaging. Studies employing adaptive graph autoencoder approaches to combine quantitative ultrasound spectroscopy, magnetic resonance imaging, and computed tomography radiomics achieved 76% sensitivity and 91% specificity for predicting radiation response in bulky nodal disease, with overall accuracy approximating 85% [57]. The diagnostic methodology involves (a) image acquisition with standardized protocols for CT, MRI, and quantitative ultrasound; (b) tumor segmentation through manual or semi-automated delineation of tumor volumes; (c) feature extraction via computational extraction of texture, shape, and intensity features; (d) feature selection using machine learning algorithms to identify most predictive features; (e) model development with training on discovery cohort with cross-validation; (f) external validation through testing on independent institutional cohorts; (g) clinical integration via development of user-friendly interfaces for clinical deployment [58,59,60,61].

Radiomics analysis predicts therapeutic response to external beam radiation in head and neck malignancies through quantitative assessment of tumor heterogeneity on pretreatment imaging. Advanced machine learning methodologies incorporating multimodal imaging data from computed tomography, magnetic resonance imaging, and quantitative ultrasound spectroscopy have demonstrated superior predictive performance compared to single-modality approaches. Recent investigations employing multiview feature selection algorithms have achieved accuracy rates exceeding 85% in distinguishing patients likely to respond favorably to definitive radiotherapy from those at high risk for treatment failure. These predictive models, which integrate radiomic signatures across complementary imaging platforms, may facilitate personalized treatment planning and enable early identification of candidates for treatment intensification or alternative therapeutic strategies [62].

### 9.4. Habitat Imaging for Neoadjuvant Response Prediction

Radiomic methodologies have demonstrated significant capability in predicting pathologic complete response to neoadjuvant chemoimmunotherapy regimens in oral cavity malignancies through sophisticated analysis of pretreatment magnetic resonance imaging. Computational habitat imaging techniques that characterize both intratumoral and peritumoral microenvironmental heterogeneity across multiple MRI sequences have achieved discrimination accuracies exceeding 85% in identifying patients likely to achieve complete pathologic response [63].

The diagnostic approach termed “habitat imaging” involves (a) multi-parametric MRI acquisition with T1-weighted, T2-weighted, diffusion-weighted, and dynamic contrast-enhanced sequences; (b) voxel-wise clustering using unsupervised algorithms to identify tumor subregions with distinct imaging characteristics; (c) habitat quantification through measurement of habitat volume, spatial distribution, and interface characteristics; (d) peritumoral analysis via extension of radiomic feature extraction to surrounding tissue; (e) integration with clinical data through combination with histologic and molecular biomarkers [64,65,66,67,68].

Peritumoral radiomic signatures appear particularly informative, potentially reflecting tumor-immune microenvironment interactions and stromal immune cell infiltration patterns that correlate with treatment sensitivity. These machine learning-derived predictive models, when integrated with conventional clinicopathologic parameters, may facilitate personalized therapeutic decision-making and enable early identification of candidates for alternative treatment intensification strategies or surgical de-escalation protocols [63].

In a study of esophageal carcinoma, enhanced CT radiomics integrated with clinical features produced combined-model AUC values of 0.914 in training and 0.859 in validation cohorts receiving chemoimmunotherapy, providing methodological precedent for response prediction workflows directly applicable to surgical planning [69].

Concordant findings have been reported in broader cohorts of locally advanced head and neck squamous cell carcinomas treated with neoadjuvant chemoimmunotherapy regimens incorporating immune checkpoint inhibitors. Multi-institutional investigations employing combined intratumoral and peritumoral radiomic signatures have consistently demonstrated superior predictive performance compared to conventional biomarkers, including programmed death-ligand 1 expression scores, achieving areas under the curve approaching 0.90 across independent validation cohorts [70].

Critical to clinical translation, these validation studies employed (a) multicenter data collection, ensuring model generalizability across different scanners and protocols; (b) standardized image preprocessing with harmonization techniques to reduce batch effects; (c) prospective validation design through testing models on temporally distinct patient cohorts; (d) decision curve analysis demonstrating clinical net benefit compared to treat-all or treat-none strategies; (e) transparent reporting with adherence to Radiomics Quality Score criteria [59,71,72,73,74,75].

These convergent results across multiple anatomic subsites and institutional datasets support the generalizability and clinical utility of magnetic resonance-based radiomic nomograms for treatment response prediction. Integration of quantitative imaging biomarkers with immunohistochemical parameters represents a promising paradigm for precision oncology in the neoadjuvant setting [70].

### 9.5. Peptide Receptor Radionuclide Therapy in Advanced Thyroid Cancers

Peptide receptor radionuclide therapy (PRRT) provides therapeutic options for advanced or dedifferentiated thyroid cancers demonstrating somatostatin receptor expression, particularly in radioiodine-refractory cases. Lutetium-177-DOTATATE binds somatostatin receptor subtype 2, achieving disease control rates of 40–60% in heavily pretreated populations. Pretreatment gallium-68-DOTATATE PET imaging enables patient selection by confirming receptor expression. Treatment-related toxicities primarily involve hematologic suppression and renal dysfunction. PRRT represents precision radiopharmaceutical therapy applicable to molecularly defined thyroid cancer subsets, providing meaningful disease control and symptom palliation when alternative options are limited [76,77].

### 9.6. Fibroblast Activation Protein Radioligand Therapy: Emerging Approach

Fibroblast activation protein (FAP) represents an emerging therapeutic target in head and neck cancers, with FAP-targeted radioligand therapy demonstrating preliminary activity in heavily pretreated populations. FAP overexpression by cancer-associated fibroblasts provides favorable therapeutic index for selective radionuclide delivery. Recent systematic reviews report encouraging disease control rates with radiolabeled FAP inhibitors conjugated to lutetium-177 or actinium-225. Diagnostic imaging using gallium-68 or fluorine-18-labeled FAP tracers enables patient selection through PET confirmation of FAP expression. Early data suggest utility in recurrent or metastatic disease refractory to conventional therapies. Critical knowledge gaps include optimal patient selection criteria, treatment protocols, and combination strategies. Despite limited availability, FAP radioligand therapy represents scientifically rational approach capitalizing on tumor microenvironment biology, with potential applicability as clinical experience accumulates [78,79,80,81].

## 10. Impact on Neoadjuvant-to-Surgery Pathways

Treatment de-intensification strategies incorporating neoadjuvant systemic therapy prior to transoral robotic surgical resection have emerged as viable approaches for managing locally advanced oropharyngeal squamous cell carcinomas. Comparative analyses utilizing propensity-matched cohorts have demonstrated oncologic equipoise between neoadjuvant chemotherapy followed by minimally invasive surgery versus immediate surgical intervention, with equivalent recurrence-free and overall survival outcomes. Notably, in human papillomavirus-associated tumors, the neoadjuvant approach facilitated adjuvant therapy omission in over half of treated patients compared to 16% in the upfront surgery cohort, attributed to downstaging and reduced pathologic risk factor prevalence following induction chemotherapy. This sequential treatment paradigm offers potential for meaningful reduction in treatment-related morbidity while preserving oncologic efficacy in carefully selected patients with HPV-driven oropharyngeal malignancies [82].

The American Society of Clinical Oncology has recognized transoral robotic surgery as a precision surgical option capable of reducing or eliminating postoperative adjuvant therapy requirements when guided by diagnostic imaging biomarkers and molecular markers in appropriately selected oropharyngeal cases [18].

However, pathological studies of post-neoadjuvant tumor regression reveal important nuances that temper enthusiasm for indiscriminate margin reduction. Regression patterns may be non-centripetal or fragmented, leaving scattered microscopic foci beyond shrunken macroscopic tumor boundaries [54]. This phenomenon necessitates integrated radiologic–pathologic planning and argues against reflexive margin narrowing based solely on clinical tumor shrinkage. Advanced diagnostic imaging with radiomics analysis may help identify these heterogeneous regression patterns preoperatively, informing surgical planning and margin determination.

## 11. Salvage Surgery: Contemporary Stratification and Outcomes

### Advanced Risk Stratification for Recurrent Disease

Management of recurrent head and neck cancer following initial therapy failure represents one of the most challenging aspects of contemporary surgical practice [33]. Recent evidence demonstrates that systematic stratification of patients can significantly improve outcomes and guide treatment decisions.

Tan et al. [83] identified initial stage IV tumors and concurrent local and regional failures as independent predictors for decreased survival after salvage surgery, with hazard ratios of 4.1 and 3.8, respectively. Using these two factors, patients could be stratified into distinct prognostic groups with two-year overall survival rates of 0%, 49%, and 83% for patients with both, one, or neither of these predictive factors, respectively.

Hamoir et al. [84] proposed another survival predictive score incorporating three independent preoperative predictors: local and regional failure, tumor site (larynx versus non-larynx), and initial stage (Stage I/II versus Stage III/IV). Patients with none, one, two, and three predictive factors had successful salvage rates of 96.2%, 62.5%, 35.5%, and 28.6%, respectively, underscoring that patients with more than one predictor factor had limited chance of successful salvage with surgery. This more accurate and easily applicable prediction model was validated in a large series of 577 patients [85].

This stratification provides a rational framework for surgical decision-making in a patient population where balance between potential benefit and surgical morbidity remains critically important. Salvage surgery offers the best survival chance for recurrent head and neck cancers, but success depends on key factors such as long disease-free interval and isolated local or regional (not combined) recurrence. Poor outcomes are linked to short recurrence time, advanced stage, positive margins, and extracapsular spread. While quality of life initially declines after surgery, it improves over a year with significant pain reduction. Future directions include combining immunotherapy with salvage surgery to improve outcomes [83,86,87].

## 12. Global Perspectives: Resource-Adapted Treatment Strategies

### Consensus Guidelines

Development of region-specific treatment guidelines represents a significant advancement in global head and neck oncology. Matos et al. [88] established 48 evidence-based recommendations for HNSCC management in resource-limited settings, addressing the reality that international guidelines cannot always be directly applied in regions with specific economic and infrastructure constraints.

The Latin American consensus emphasized several key principles: prioritizing CT scanning over MRI for locoregional staging due to greater availability and lower cost; implementing sentinel lymph node biopsy only when comprehensive technical resources are available, otherwise defaulting to selective neck dissection; and establishing clear criteria for adjuvant therapy based on available treatment modalities. Key surgical recommendations included margins >5 mm for oral, oropharyngeal, supraglottic, and hypopharyngeal tumors, with >1 mm considered adequate for glottic tumors. The consensus emphasized that close margins (1–5 mm) should not be considered positive margins, providing important guidance for treatment planning in resource-constrained environments. Strong evidence supports adjuvant concurrent chemoradiation for patients with positive surgical margins and/or metastatic lymph nodes with extranodal extension, with alternative dosing regimens including weekly cisplatin (40 mg/m^2^) providing options for patients who may not tolerate intensive protocols [88].

## 13. Quality Improvement and Outcome Measurement

### Development of Specialty-Specific Metrics

Recognition of unique challenges in head and neck surgical oncology has led to development of specialty-specific quality improvement programs. Creation of a National Surgical Quality Improvement Program (NSQIP) focused on head and neck oncology represents a significant advancement in outcome measurement and quality assessment, demonstrating feasibility of capturing 100% of complex head and neck reconstruction cases [89].

This initiative incorporates disease- and procedure-specific variables, including TNM staging, reconstruction type, and detailed functional outcomes such as feeding tube and tracheostomy dependence. Contemporary quality improvement data reveal that only 13.5% of patients undergoing complex head and neck reconstruction have no preoperative risk factors, with 43.6% presenting with three or more risk factors. Despite this complexity, 30-day mortality rates of 1.0% and acceptable morbidity profiles suggest that systematic approaches to quality measurement can support outcome optimization [89].

## 14. Emerging Technologies and Future Directions

### Artificial Intelligence and Machine Learning Applications

Artificial intelligence (AI) and machine learning technologies are beginning to exert meaningful influence on head and neck cancer management through multiple applications [90,91]. Diagnostic assistance systems represent one of the most promising near-term applications, with potential to enhance tumor detection accuracy and provide sophisticated prognostic modeling capabilities that support clinical decision-making. Surgical planning optimization through AI analysis of imaging data promises to improve treatment selection and reduce complications by providing detailed anatomical analysis and outcome prediction based on large datasets [92].

## 15. Precision Medicine Integration: Future Directions for Diagnostic Technologies

The convergence of molecular diagnostics, advanced surgical techniques, and quality improvement initiatives creates opportunities for increasingly personalized surgical care. While current genomic profiling and imaging biomarkers have established utility in treatment selection, future refinement will focus on dynamic biomarker monitoring throughout treatment courses, enabling real-time adaptation of surgical strategies.

Understanding tumor biology, immune microenvironment characteristics, and therapeutic response patterns through longitudinal sampling may enable more precise surgical planning and treatment sequencing decisions. Identification of PD-L1 combined positive score as a predictor of immunotherapy response exemplifies the potential for molecular guidance in treatment selection [11]. However, additional diagnostic markers will be needed to refine existing protocols for individual patient adaptation and disease aggressiveness assessment.

Critical questions remain, including optimal immunotherapy cycle numbers, treatment duration, and identification of patients who may benefit from treatment de-escalation or intensification based on evolving molecular profiles during therapy. Future diagnostic platforms will likely integrate (a) liquid biopsy technologies with serial circulating tumor DNA monitoring for minimal residual disease detection; (b) advanced imaging biomarkers with functional and molecular imaging for non-invasive tumor characterization; (c) immune monitoring through peripheral blood immune profiling to predict treatment response; (d) multi-omic integration via combined genomic, transcriptomic, proteomic, and metabolomic analyses; (e) artificial intelligence platforms using machine learning algorithms integrating diverse data types for individualized risk stratification [93,94,95,96,97,98,99,100,101,102].

Standardization of these diagnostic methodologies across institutions, with rigorous validation in diverse patient populations, will be essential for equitable implementation of precision medicine approaches.

## 16. Current Challenges and Future Integration

Despite significant advances in surgical techniques, imaging technologies, and systemic therapies, important challenges persist in head and neck cancer management. Lack of high-quality randomized comparative data for many clinical scenarios contributes to treatment variability based on institutional preferences and available expertise. Healthcare system disparities in access to advanced surgical techniques, diagnostic technologies, and multidisciplinary care represent important challenges for achieving optimal outcomes across diverse patient populations.

**Figure 2:**
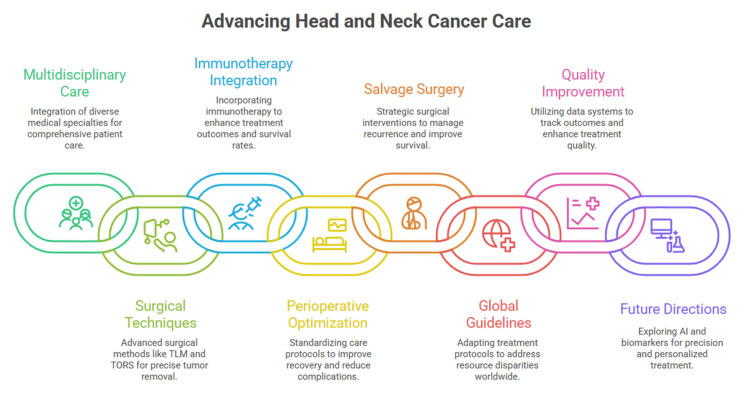
Advancing head and neck cancer care through validated diagnostic technologies and multidisciplinary integration.

Implementation of advanced surgical techniques and diagnostic platforms requires significant institutional investment in equipment, training, and ongoing education that may limit widespread adoption. Despite challenges represented by high instrumentation costs for robotic systems and genomic profiling platforms, progressive technological advancement and increasing utilization may reduce these expenses over time, facilitating broader access to minimally invasive surgical approaches and precision diagnostics (Figure 2).

Additional challenges include (a) standardization of diagnostic assays, as variability in methodologies across institutions limits comparability; (b) validation in diverse populations, since most diagnostic biomarkers have been validated primarily in specific demographic groups; (c) integration into clinical workflows, as practical implementation of complex diagnostic algorithms requires user-friendly interfaces; (d) reimbursement and access, with economic barriers limiting availability of advanced diagnostics in many healthcare systems; (e) regulatory frameworks, as approval pathways for complex diagnostic platforms remain evolving [103,104,105,106].

This narrative review contains inherent methodological limitations. Unlike systematic reviews employing structured search protocols, narrative reviews involve selective citation based on author judgment, potentially introducing selection bias. Concentration on recent publications may underrepresent landmark historical studies, and predominantly English-language literature from high-resource settings may overlook contributions from diverse healthcare contexts. Rapid evolution of precision medicine technologies creates temporal limitations, as evidence published during manuscript preparation may not be incorporated. Heterogeneity in outcome reporting across studies limits direct comparisons and precludes quantitative meta-analysis. The review’s scope deliberately focuses on adult squamous cell carcinomas of oral cavity, oropharynx, and larynx, omitting pediatric malignancies and rare histologies. Absence of formal quality assessment represents additional limitation. Despite these constraints, narrative reviews provide valuable synthesis of complex, rapidly evolving fields where systematic methodologies may prove impractical

## 17. Conclusions

The role of surgery in head and neck cancer management has undergone fundamental transformation, evolving from radical organ-sacrificing procedures toward integrated, function-preserving approaches that prioritize both oncologic control and quality of life within diverse global healthcare contexts. This evolution reflects advances in surgical technique, improved understanding of tumor biology through validated diagnostic biomarkers, successful integration of perioperative chemotherapy and immunotherapy protocols, implementation of comprehensive genomic profiling and radiomics-based predictive models, and recognition of the importance of multidisciplinary care coordination in achieving optimal patient outcomes.

Technological advances culminating in minimally invasive surgical techniques, including transoral robotic surgery and transoral laser microsurgery, have expanded therapeutic options while reducing treatment morbidity and improving functional preservation. Development of evidence-based guidelines has provided comprehensive frameworks for patient selection and implementation, ensuring optimal outcomes while maintaining safety standards. Most significantly, integration of validated diagnostic biomarkers with neoadjuvant and adjuvant immunotherapy represents the most transformative recent advancement, with the KEYNOTE-689 trial establishing perioperative pembrolizumab as a new standard of care for advanced stage oral, p16-negative oropharyngeal, hypopharyngeal, and laryngeal cancer without compromising surgical completion rates [11,107].

Integration of comprehensive genomic profiling platforms, refined HPV detection methodologies, and validated radiomics-based predictive models has fundamentally reshaped surgical oncology decision-making. These diagnostic technologies enable precision patient selection for neoadjuvant therapy, inform surgical extent and technique, and guide adjuvant treatment decisions with unprecedented granularity. Tumor mutational burden assessment serving as a validated predictive biomarker, immune microenvironment characterization through multiplex immunohistochemistry, and radiomics-based response prediction achieving diagnostic accuracies exceeding 85% in validation studies provide concrete frameworks for distinguishing patients most likely to benefit from specific therapeutic approaches. The multicenter NECTORS experience demonstrating reduced distant metastasis rates with neoadjuvant-surgery pathways exemplifies the potential of biomarker-integrated treatment sequencing.

The central role of diagnostic technologies in achieving precision surgery and personalized treatment represents the defining characteristic of contemporary head and neck oncology. Comprehensive genomic profiling platforms identifying actionable alterations in over 90% of cases, PD-L1 combined positive score serving as a validated predictive biomarker for immunotherapy selection, and radiomics-based models achieving areas under the curve approaching 0.90 for treatment response prediction demonstrate the transformative potential of validated diagnostic methodologies. These technologies enable (a) accurate patient stratification, distinguishing responders from non-responders before treatment initiation; (b) response-adaptive therapy, modifying treatment intensity based on objective biomarker assessments; (c) organ preservation strategies, identifying candidates for surgical de-escalation without compromising oncologic outcomes; (d) salvage surgery optimization, selecting patients most likely to benefit from aggressive interventions; (e) clinical trial eligibility, matching patients to appropriate investigational therapies.

Improvements in both oncologic and functional outcomes achieved through multidisciplinary tumor board implementation, coupled with evidence-based risk stratification for salvage surgery and resource-appropriate treatment protocols, demonstrate the potential for truly personalized cancer therapy that optimizes survival while preserving quality of life. Contemporary evidence supports systematic quality improvement initiatives that enable specialty-specific outcome measurement and continuous improvement in care delivery.

However, important limitations persist in diagnostic implementation. Variable assay performance across racial and ethnic populations mandates confirmatory testing protocols and deliberate inclusion of diverse cohorts in validation studies. Real-world constraints in drug availability, clinical trial access, and institutional resources limit translation of genomic insights into therapeutic interventions. Many imaging biomarkers remain institutionally validated rather than standardized across centers, and prospective randomized evidence demonstrating survival benefits from biomarker-driven surgical strategies remains limited or is still evolving.

Future progress in head and neck cancer surgery fundamentally depends on continued refinement and validation of diagnostic technologies. Standardization of genomic profiling platforms, radiomics methodologies, and immune biomarker assays across institutions will be essential for equitable implementation. Development of user-friendly clinical decision support systems integrating multimodal diagnostic data will facilitate adoption in diverse practice settings. Most critically, prospective randomized trials demonstrating that diagnostic biomarker-guided treatment strategies improve survival and functional outcomes compared to standard approaches remain essential for establishing true clinical utility.

As prospective validation studies mature and implementation frameworks become standardized, the paradigm shift from radical resection toward biomarker-integrated precision surgery promises to optimize both oncologic control and functional preservation. The surgical renaissance in head and neck oncology reflects not a diminishment of surgical importance, but rather its evolution into a more sophisticated component of personalized cancer therapy guided by validated diagnostic technologies. This transformation requires continued collaboration across molecular pathology, radiology, surgical oncology, medical oncology, and data science disciplines to ensure equitable access to these transformative diagnostic approaches and to realize their full potential in improving patient outcomes while accommodating diverse global healthcare environments and maintaining uncompromising standards for both oncologic control and functional preservation.

## Figures and Tables

**Table 1:** Multidisciplinary Team Members and Their Primary Responsibilities.

Team Member	Primary Responsibilities
Head and Neck Surgical Oncologist	Surgical candidacy determination Timing coordination Tumor resection and neck dissection Reconstruction planning (with plastic surgeon) Surgical complication management
Radiation Oncologist	Radiation therapy design and delivery Dose determination Adjuvant/definitive protocols Radiation toxicity management
Medical Oncologist	Systemic chemotherapy, target therapy and immunotherapy Perioperative therapy Clinical trial enrollment Systemic treatment toxicity management
Pathologist	Diagnosis including molecular analysis Margin and lymph node assessment Biomarker testing
Radiologist	Diagnostic imaging interpretation Radiomics analysis Resectability assessment Response evaluation
Speech-Language Pathologist	Swallowing and speech assessment and management Rehabilitation therapy
Nutritionist	Nutritional status evaluation; Feeding support coordination; Nutritional treatment-related complication management
Palliative Care Specialist<	Pain and symptom control; Psychosocial support; Goals-of-care discussions
Social Worker	Care coordination Psychosocial barrier management Resource connection
Nurse Navigator	Primary contact Patient/family education; Appointment coordination Treatment monitoring

## Data Availability

No new data were created or analyzed in this study.
